# Complete Genome Sequence and Methylome Analysis of Sphaerotilus natans subsp. *sulfidivorans* D-507

**DOI:** 10.1128/MRA.01194-19

**Published:** 2019-11-14

**Authors:** Alexey Fomenkov, Margarita Grabovich, Elena Belousova, Dmitry Smolyakov, Galina Dubinina, Richard J. Roberts

**Affiliations:** a New England BioLabs, Inc., Ipswich, Massachusetts, USA; b Department of Biochemistry and Cell Physiology, Voronezh State University, Voronezh, Russia; c Federal Research Centre “Fundamentals of Biotechnology,” Russian Academy of Sciences, Moscow, Russia; Portland State University

## Abstract

Sphaerotilus natans subsp. *sulfidivorans* D-507 is an environmental isolate from a sulfate spring in the northern Caucasus region of Russia. This heterotrophic bacterium is involved in the oxidation of reduced sulfur derivatives. This report includes the finished genome of this strain. In addition, we provide methylation data and the associated enzymes predicted to be responsible for each modified motif.

## ANNOUNCEMENT

Sphaerotilus natans subsp. *sulfidivorans* strain D-507 was previously described (1) and deposited into the DSMZ collection under DSM 22546. Native genomic DNA (10 μg) of S. natans subsp. *sulfidivorans* strain D-507 was obtained directly from the DSMZ collection. The Escherichia coli genomic DNAs from strains ER2796 and ER3081 with cloned methyltransferase genes were purified using the Monarch genome purification kit (New England BioLabs, Ipswich, MA).

Single-molecule real-time (SMRT) libraries were sequenced using the Pacific Biosciences (PacBio) RS II sequencing platform. Briefly, SMRTbell libraries were constructed from a genomic DNA sample sheared to ∼10 to 20 kb using the g-Tube protocol (Covaris, Woburn, MA), end repaired, and ligated to PacBio hairpin adapters. Incompletely formed SMRTbell templates and linear DNAs were digested with a combination of exonuclease III and exonuclease VII (New England BioLabs). DNA qualification and quantification were performed using a Qubit fluorimeter (Invitrogen, Eugene, OR) and 2100 Bioanalyzer (Agilent Technology, Santa Clara, CA). A SMRTbell library was prepared according to the PacBio sample preparation protocol, which included additional separation on a BluePippin system (Sage Science, Beverly, MA), and sequenced with C4-P6 chemistry, using 2 SMRT cells, one with a non-size-selected 8-kb library and one with a size-selected 11-kb library, and a 360-minute collection time. The data from the two cells were combined, resulting in 163,482 sequencing reads with a 7,894-bp mean subread length, yielding 2.2 Gb, that were *de novo* assembled using Hierarchical Genome Assembly Process 3 (HGAP.3) version 2.3.0 with default quality and read length parameters and then polished 3 times using Quiver (2). The polished assembly generated 3 closed circular genome elements with a 69.93% GC content for the main chromosome (3,983,768 bp), a 70.48% GC content for plasmid pSna507_unt10 (303,297 bp), a 63.79% GC content for plasmid pSna507_unt12 (84,154 bp), and one linear contig with a GC content of 67.75% for pSna507_unt13 (59,773 bp). The assembled sequence was annotated using the NCBI Prokaryotic Genome Annotation Pipeline (PGAP) (3, 4).

One advantage of the PacBio sequencing platform is its ability to detect the epigenetic state of sequenced DNA (4–7). Nine modified DNA motifs were detected by SMRT motif and modification analysis version 2.3.0. Eight motifs contain m6A modifications, and one contains an m4C motif on native DNA. Genome scanning with the SeqWare program predicted eight methyltransferase genes and one Brex2 system in the genome. Five type I methyltransferases and their corresponding S genes and one type IIG system, M.Sna507II, have been successfully cloned into the pACYC184 vector under the control of the *tet* promoter into nonmethylated ER2796, and another type IIG gene, Sna507ORF3475P, was cloned into the pSAPV6 vector under the control of the T7 promoter into ER3081. All E. coli clones were resequenced in SMRT cells to detect the individual modification motifs. Correlations between 7 modified motifs and their methyltransferase genes were determined experimentally. The last m6A motif belongs to M.Sna507VII, and the orphan m4C motif belongs to a Brex2 system. Thus, all observed motifs were matched with their responsible methyltransferases through cloning and expression in E. coli. The results are shown in Table 1 and have been deposited in REBASE (8).

**TABLE 1:** Summary of genome elements, methyltransferase genes, and their motifs identified in Sphaerotilus natans subsp. *sulfidivorans* strain D-507

Genetic element of Sphaerotilus natans subsp. *sulfidivorans* strain D-507	GenBank accession no.	Genome size (bp)	Genome coverage (×)	Methylase (RM^*e*^ system) name	Recognition motif^*a*^	Methylation RM^*e*^ type
Chromosome	CP035708	3,983,768	203.71	M.Sna507I^*d*^	CGC**A**N_7_**T**GCG	m6A, I
				M.Sna507II^*d*^	GAGA**A**G	m6A, IIG
				M.SnaIII^*d*^	CGC**A**N_7_C**T**GC	m6A, I
				M.SnaIV^*d*^	CGG**A**N_6_**T**GG	m6A, I
				S.Sna507V^*b*^^,^^*d*^	CC**A**GN_7_**T**CGA	m6A, I
				M.Sna507VII	GC**A**GN_7_R**T**RTC	m6A, I
				Sna507ORF3475P^*d*^	CRTTG**A**G	m6A, IIG
Plasmid pSna507_unt10	CP035710	303,297	144.51			
Plasmid pSna507_unt12	CP035709	84,154	112.84	Sna507ORF18450^*d*^	Inactive	m6A, I
				M.Sna507VI^*c*^^,^^*d*^	CG**A**GN_5_CA**T**C	
Linear contig pSna507_unt13	CP035707	59,773	103.08	M.Sna507ORF65P	CCCG**C**CC	m4C, Brex2 system

a Modified bases, or the base opposite to them, are boldface and underlined boldface, respectively.

b This system has no separate methylase gene, and so the S subunit must combine with a methylase subunit from a different system.

c This system requires the S1 subunit to determine specificity.

d MTase genes cloned and expressed in E. coli strain ER2796 (9) and ER3081.

e RM, restriction modification.

### Data availability.

The complete genome sequences of Sphaerotilus natans subsp. *sulfidivorans* strain D-507 and its plasmids are available in GenBank under the accession numbers CP035708, CP035710, CP035709, and CP035707. Original sequence reads from the individual SMRT cells have been deposited in the NCBI database under SRA accession numbers SRR8539625 and SRR8539624 with BioProject accession number PRJNA520894.
